# The relationship between *DRD4* polymorphisms and phenotypic correlations of behaviors in the collared flycatcher

**DOI:** 10.1002/ece3.1041

**Published:** 2014-03-24

**Authors:** László Z Garamszegi, Jakob C Mueller, Gábor Markó, Eszter Szász, Sándor Zsebők, Gábor Herczeg, Marcel Eens, János Török

**Affiliations:** 1 Department of Evolutionary Ecology, Estación Biológica de Doñana-CSICSeville, Spain; 2 Department of Behavioral Ecology and Evolutionary Genetics, Max Planck Institute for OrnithologySeewiesen, Germany; 3 Behavioural Ecology Group, Department of Systematic Zoology and Ecology, Eötvös Loránd UniversityBudapest, Hungary; 4 Department of Plant Pathology, Corvinus University of BudapestBudapest, Hungary; 5 Ecology Research Group, Hungarian Academy of Sciences, Hungarian Natural History MuseumBudapest, Hungary; 6 Université Paris-Sud, Centre de Neurosciences Paris-SudUMR 8195, Orsay, France; 7 Ethology Group, Department of Biology, University of AntwerpWilrijk, Belgium

**Keywords:** Antipredator behavior, behavioral genetics, behavioral syndromes, linkage disequilibrium, novelty seeking, personality, temperament

## Abstract

There is increasing evidence that the genetic architecture of exploration behavior includes the dopamine receptor D4 gene (*DRD4*). Such a link implies that the within-individual consistency in the same behavior has a genetic basis. Behavioral consistency is also prevalent in the form of between-individual correlation of functionally different behaviors; thus, the relationship between *DRD4* polymorphism and exploration may also be manifested for other behaviors. Here, in a Hungarian population of the collared flycatcher, *Ficedula albicollis*, we investigate how males with distinct *DRD4* genotypes differ in the consistent elements of their behavioral displays during the courtship period. In completely natural conditions, we assayed novelty avoidance, aggression and risk-taking, traits that were previously shown repeatable over time and correlate with each other, suggesting that they could have a common mechanistic basis. We identified two single-nucleotide polymorphisms (SNP554 and SNP764) in the exon 3 of the *DRD4* gene by sequencing a subsample, then we screened 202 individuals of both sexes for these SNPs. Focusing on the genotypic variation in courting males, we found that “AC” heterozygote individuals at the SNP764 take lower risk than the most common “AA” homozygotes (the “CC” homozygotes were not represented in our subsample of males). We also found a considerable effect size for the relationship between SNP554 polymorphism and novelty avoidance. Therefore, in addition to exploration, *DRD4* polymorphisms may also be associated with the regulation of behaviors that may incur fear or stress. Moreover, polymorphisms at the two SNPs were not independent indicating a potential role for genetic constraints or another functional link, which may partially explain behavioral correlations.

## Introduction

Unraveling the extent by which behavior is determined by genetic makeup could be vital to understanding the evolutionary ecology of behavioral phenotypes within the normal range of variation in different taxa (van Oers and Mueller 2010; Tschirren and Bensch 2010). Natural populations of animals are particularly important in this research agenda, because they are less influenced than human societies by socio-ecological and cultural effects that hamper our ability to attribute mechanisms to natural selection (Cronk 1991 for behavioral ecology in general; Fidler et al. 2007 for behavioral genetics in particular). For example, specific genotype–environment interactions, the complex population genetic structure, and learning processes are typical confounders to consider, especially in humans. Moreover, the fact that human societies preserve extreme phenotypes due to obvious ethical reasons through medication and hospitalization makes it difficult to speculate about the consequences for individual fitness. Accordingly, a good number of studies reported gene–behavior associations focusing on a wide range of behavioral and molecular traits in nonhuman models including fishes (e.g., Boehmler et al. 2007; St-Cyr and Aubin-Horth 2009; Filby et al. 2010; Lorenzi et al. 2012; Laine et al. 2014), birds (e.g., Tlemcani et al. 2000; Vignal et al. 2005; Cheng and Muir 2007; Mueller et al. 2011), and mammals (e.g., Brodkin et al. 2002; Del Punta et al. 2002; van der Veen et al. 2006; Yan et al. 2013).

A striking recognition of recent day's evolutionary biology is that, although one would expect that individuals flexibly adjust their behaviors depending on immediate environmental conditions, consistent between-individual variation in many behaviors is maintained in natural populations of animals (Dingemanse et al. 2010; Sih et al. 2012). This consistency may be prevalent as the repeatability of the same behavior over different time and contexts (known as “personality”), but also as the correlation between functionally different behaviors (known as “behavioral syndrome”) (Dingemanse and Wolf 2010; Garamszegi and Herczeg 2012; Jandt et al. 2014). The genetic basis of such consistent phenotypic variation and its relevance for reproductive success and survival are yet to be pinpointed in most of the cases (Dingemanse and Wolf 2010). Wider knowledge about the molecular mechanisms that drive behaviors to vary at an individual-specific manner can be important for at least two reasons. First, a functional linkage between genes and behavioral phenotypes can imply a role for a mechanistic constraint that sets up an intrinsic limit for behavioral flexibility. A common inner control of behaviors results in the consistent variation of the same behavior across time and context and also in the nonindependence of functionally different behaviors (Sih et al. 2004; Bell 2005). For example, if behaviors are linked due to pleiotropic genes that simultaneously and directly control several traits, they are hard to uncouple on an evolutionary timescale. On the other hand, if correlating behaviors governed by separate, independent mechanism (e.g., environmental constraints) behaviors can be easily detached. Second, known forms of balancing selection acting on genetic polymorphisms, such as overdominance and antagonistic pleiotropy, may help forming explanations for phenotypic variations in natural populations (van Oers and Mueller 2010).

As revealed by a list of both the genome-wide and the candidate-gene approaches (van Oers and Mueller 2010), one of the potential genes that contributes to the government of consistent behaviors is the dopamine receptor D4 (*DRD4*) gene. The gene product is an important component of the dopaminergic system, and its function is linked to many neurological and psychiatric disorders but also contributes to the guidance of normal behaviors such as novelty seeking in humans, although some evidence is at conflict with this general suggestion (Kluger et al. 2002; Schinka et al. 2002; Reif and Lesch 2003; Ebstein 2006). Moreover, recent studies in other model species, such as different mammals and birds, revealed that polymorphism in the *DRD4* gene is associated with novelty seeking, exploration, and escape behavior (Momozawa et al. 2005; Mogensen et al. 2006; Bailey et al. 2007; Fidler et al. 2007; Hejjas et al. 2007; Gutierrez-Gil et al. 2008; Kluen et al. 2012). Passerine birds appear particularly important targets for such research, as high degree of mutation rate and codon bias indicates that this genomic region is under strong selection (Abe et al. 2011). Some of these studies have already addressed questions about how genetic polymorphism mediates individual mean behavioral phenotypes (Fidler et al. 2007; Korsten et al. 2010; Kluen et al. 2012; Carvalho et al. 2013; Mueller et al. 2013). However, the genetic background of consistency that is manifested at the between-trait context remained undetermined.

The collared flycatcher (*Ficedula albicollis*) is a small hole-nesting European passerine that has been intensively studied for consistent behaviors of males during the courtship period. Based on the repeated measures of the same individuals in different days in their natural environment, it seems evident that displaying birds demonstrates repeatable singing, territorial and risk-taking behavior over time (Garamszegi et al. 2004, 2006, 2012b). Moreover, such consistent variation is also revealed across functionally different behaviors, as shown by their statistical correlation (Garamszegi et al. 2008, 2009b). Some of these behaviors are known to have fitness consequences, as they are related to mating success and the ability to retain territories (Garamszegi et al. 2004, 2006, 2008). Such knowledge in combination with the advantages of the noninvasive methods used to characterize behaviors without causing any disturbance to the animals makes this system ideal to determine the possible genetic underpinning of behavioral differences by using a genetic marker.

The objective of this study was, therefore, to establish a link between genetic polymorphism and correlation between functionally different behaviors in collared flycatchers by using a candidate gene approach and by focusing on the *DRD4* gene. We characterized novelty avoidance, aggression, and risk-taking displayed during the courtship behavior of males in the vicinity of their nest box. Birds were only captured after being monitored for their behaviors, when the sampling for the genetic analyses took place. We identified two *DRD4* exon 3 polymorphisms, which were used to describe individual genotypes. Based on previous results found in other passerines (Fidler et al. 2007; Korsten et al. 2010; Kluen et al. 2012), we predicted behavioral differences between particular genotypes. Moreover, we predicted that if behaviors are correlating because they have a common genetic control, different behaviors would show similar associations with *DRD4* genetic polymorphism.

## Methods

### General field methods

The field work was carried out in a Hungarian population of the collared flycatcher that breeds in the Pilis Mountains close to Budapest, Hungary (47°43′N, 19°01′E), where about 800 artificial nest boxes had been positioned in the eighties to allow field studies of hole-nesting passerines (Török and Tóth 1988). As a part of a long-term population study on behavioral consistency, we collect standard behavioral data in each breeding season during the courtship period since 2007. For the current investigation, we used information on three male display traits in relation to genetic variability at the *DRD4* from three consecutive seasons between 2008 and 2010. According to the established field protocols, around the expected date of the first males returning from the wintering sites, we regularly visited the field site for newly arrived, territorial but unpaired males showing the typical courtship displays (nest-box presentation and singing). Once these males were localized at a known nest box, we initiated our behavioral trials (see below) at the same locality allowing measurements on free individuals. We captured males only after these assays with a conventional nest-box trap, which was followed by the measurements of their morphological traits (such as body mass with a caliper to a nearest 0.1; tarsus length and the size of the white forehead and wing patches with a ruler with nearest 0.1 mm). We also assigned males into a two-scale age category (yearlings/older), based on the typical plumage coloration (males in their first breeding year have brown remiges, while males from their second year wear black remiges). We finally took a blood sample from the brachial vein for the subsequent molecular analyses (see below). After the measurements and blood sampling, birds were released. Most of the released birds (i.e., 50–70% depending on year) were observed continuing their courtship activity and realizing successful breeding (see ethical note in Garamszegi et al. 2009b).

### Behavioral measurements

The detailed protocols of the behavioral assays have repeatedly been described elsewhere (Garamszegi et al. 2008, 2009b, 2012b). Briefly, we characterized three behavioral traits during the most active morning period (usually between 6.00 and 12.00 h) for each individual. First, we estimated novelty avoidance by timing the latency needed to resume courtship activity in the presence of a novel object. In this test, we defined baseline courtship activity in the presence of a caged stimulus female (placed on top of the nest box), as the time interval that elapsed until the male landed on the entrance hole of his nest box (a typical element of the courtship display) after appearing on its territory. Then, we mounted a novel object (white A6 sheet with small spots of variable colors) and measured the same variable in the same conditions. Novelty avoidance, therefore, can be calculated as a difference between the two latency scores measured in the two situations (i.e., males increasing their latency to the first visit of the nest box's hole in the presence of a novel object can be considered as novelty avoiders).

Secondly, we estimated aggression. Following the novelty avoidance test, we presented the resident male with a live and caged decoy of the same sex to stimulate territorial intrusion. With this stimulus, we exerted aggressive behavior from the territory owner, which consists of several behavioral elements (Garamszegi et al. 2006). For simplicity as well as for ethical reasons, and also because it showed strong correlations with the other variables measured during longer tests, we chose the latency to the first attack (the time elapsed between the appearance of the resident on the territory and the event when it first landed on the cage of the decoy bird) to describe aggression. Accordingly, males attacking the intruder immediately can be considered as aggressive males.

Thirdly, we assessed risk-taking based on a slight modification of a protocol that has been developed for measuring flight initiation distance, a widely used comparative metric of wariness (Blumstein 2003). In our design, we waited until the focal male in the above experiment became engaged in a territorial dispute with the stimulus bird. Once it was localized on the top of the decoy's cage, the observer started to move toward the birds with a normal walking speed. When the resident interrupted its aggressive activity due to the recognition of the risky situation (i.e., presence of a potentially predator in the vicinity) and fled away, the observer stopped walking. Then, the observer waited again until the resident returned to fight again on the top of the decoy's cage, and when it happened (i.e., most of the cases), s/he continued the approach. This was repeated until the resident did not return to the cage anymore within at least one minute. In this situation, the distance between the decoy and the last standing point of the observer was measured as the number of steps of approximately one meter to reflect flight initiation distance. We developed this sequence to eliminate the confounding effect of very aggressive males not noticing the approaching human. In our protocol, by allowing the focal male to return, we were certain than it had noticed the observer and thus perceived the situation risky (most of the birds emitted alarm calls). Consequently, we treated males that demonstrated small flight initiation distance as risk-takers.

Although we hold records for these behaviors for 7 years, for the present study, we focused on 3 years from the overall dataset, for which we also have genetic data (one individual was included only once in the analyses). For some analyses that did not require information on *DRD4* genotypes (e.g., for testing the correlation between morphology and behavior, or between different behavioral traits), we relied on the entire dataset. Although, we aimed at measuring all behavioral traits in all males, due to various constraints, information on particular behaviors may not be available in few cases causing slight variation in sample size among analyses (i.e., for some males, information on aggression and/or risk-taking may not be available). Individuals assayed in the behavioral tests and screened for DRD4 were not used in other experiments.

### Genetic analyses

**Figure 1:**
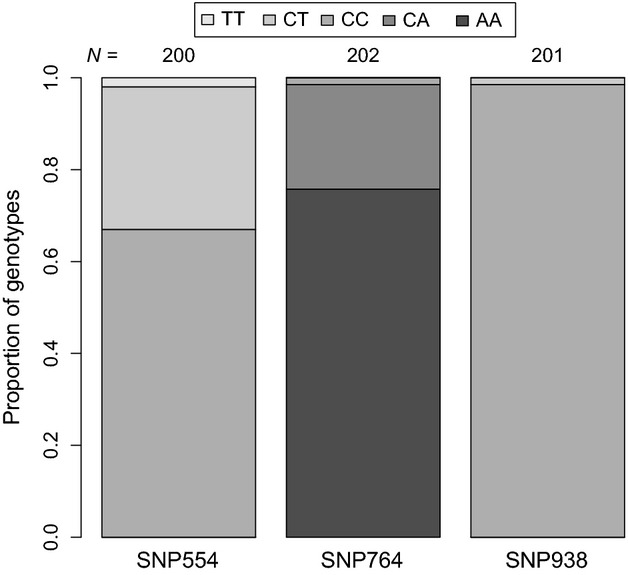
Proportions of *DRD4* genotypes at three SNPs (SNP554, SNP764, and SNP938) in a Hungarian collared flycatcher population (sexes are pooled).

Altogether, over the 3 years, we collected blood samples for *DRD4* screening for more than two hundred individuals (see sample sizes in Fig. 1 and also in the text of the “Results” section). This sample included both males (*N* = 116) and females (*N* = 86) from the courtship as well as from the chick-feeding period in order to obtain an unbiased picture about the frequency distribution of genotypes. However, when exploring the potential relationship between behavior and polymorphism at the *DRD4*, we focused on a subsample of males from the courtship period with information on the three aforementioned behavioral traits.

The blood samples collected in the field were conserved in a Queens buffer. After returning from the daily field trips, these were stored in a fridge (at 4°C) until the end of the field season. Then, in the laboratory, we amplified and directly sequenced the complete exon 3 of the *DRD4* (604 bp) in ten randomly selected flycatcher individuals using the primers DRD4_I2F (CACCACACCAGGACTGACT) and DRD4_I3R (GTGKGACAAGSTGGCACATTT). We identified three loci within this region, one of which turned out to be little variable (SNP937, minor allele frequency <1%, Fig. 1). The remaining two SNPs (SNP554, SNP764) were used in the association analysis. All loci were synonymous coding. The ABI PRISM SNaPshotTM Multiplex Kit (Pati et al. 2004) was used with the extension primers CCCAGTCATATTTGGCCTCAA (SNP554), GACTGACTGACTGGAAGCTGTATCACCCCCC (SNP764), and GACTGACTGACTGACTGACTCAAGCGGGCCAAGATCAACGG (SNP937) to genotype all individuals for these SNPs blindly to the identity of birds and their behavioral scores. Multiple samples for the same individuals provided identical screen results for the *DRD4* genotypes. The PCR protocol is given in Mueller et al. (2011).

Given our experience with gene-wide (>10 kb) *DRD4*-exploration associations in the great tit, *Parus major* (Mueller et al. 2013), we infer that the selected target region (∼ 600 bp comprising the complete DRD4 exon 3) lies in the center of the associated homologous great tit region. Furthermore, the great tit-specific exon 3 SNP830 was among the most significantly associated SNPs in the investigated populations, in which a study-wide association was found. Our target region in flycatchers therefore likely serves as a marker (or functional) region for potential *DRD4* associations.

### Statistics

**Figure 2:**
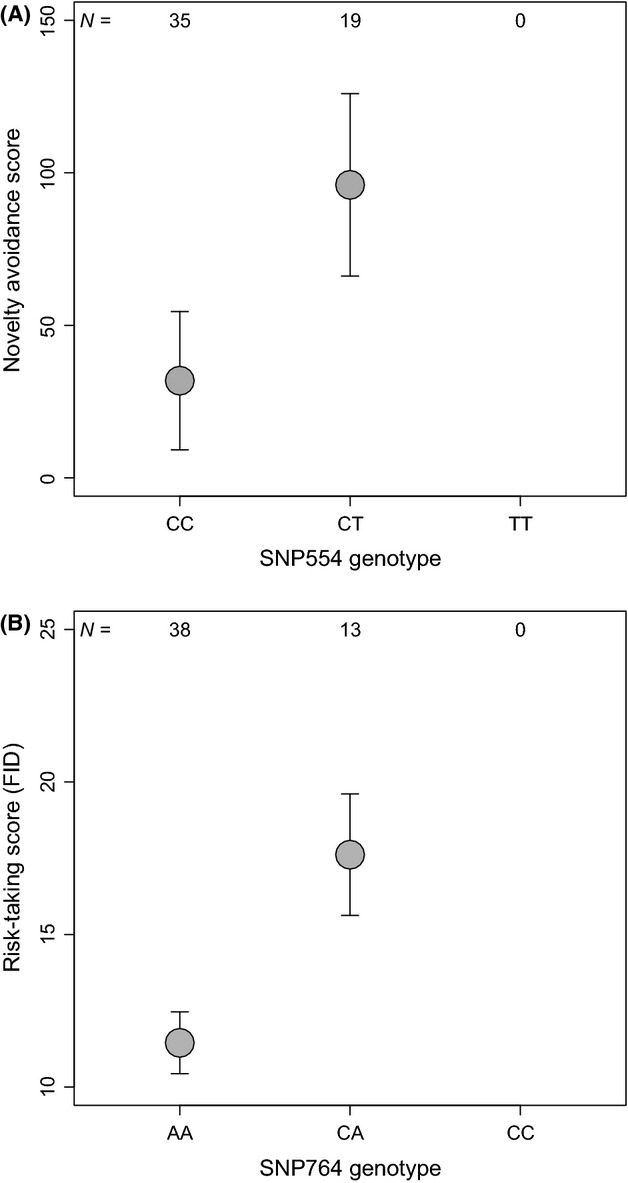
(A) Novelty avoidance scores (latency – in sec – to land on the entrance hole of the nest box in the presence of a novel object relative to the same latency measured in the absence of novelty) in males of the collared flycatchers during their courtship activity in association with *DRD4* SNP554 genotype. (B) The relationship between estimates of risk-taking (flight initiation distance – in meter – in the presence of a potential predator) and SNP764 genotype. Figures show group-specific means (gray circles) and standard errors (bars). The corresponding statistics (when potentially confounding variables are held constant) are given in Table 2.

Before entering them into any analyses, the distribution of continuous variables was investigated graphically. If these figures indicated strongly skewed distributions for the predictor variables (see model construction below), we applied log_10_-transformation to obtain more or less symmetrical distribution (e.g., normal or uniform as required for linear modeling) that avoids having more influential values in one end of the distribution than in the other end. The response variables (behavioral traits) were either log_10_-transformed (risk-taking), or left untransformed (aggression, novelty avoidance), but models were also run based on their rank-transformed values as well as based on bivariate categories (see details below). Each individual was represented with a single measurement (see the repeatability of traits based on repeated measurements within season in Garamszegi et al. 2012b). The date of the behavioral observation was standardized among years by defining day 1 in each season based on the date when the first males were seen on the field site. For the categorical predictors, most importantly for the *DRD4* genotypes, we drew frequency diagrams to see the number of cases within each group (Fig. 1). This diagnostics revealed that some genotypes (e.g., “TT” for SNP554 and “CC” for SNP764) are extremely rare, which incurs the statistical risk of having cases within rare levels that are disproportionately more influential than cases in more common levels. As a solution, one can drop these rare genotypes from the data prior to the analyses instead of defining additional factors based on few observations. However, the subsample of males with information on both behavioral and genetic traits did not include these rare genotypes, thus the corresponding model necessarily relies on two-state variables for these predictors (Fig. 2).

We used chi-square test to compare the frequency distribution of genotypes with expectations derived from the Hardy–Weinberg theorem and also to compare genotype frequencies between sexes as well as for the association between the two SNPs.

To analyze the relationship between behavioral phenotypes and *DRD4* polymorphism, we created general linear mixed models with the following structure. In our starting models, the measured behavioral variables were treated as response variables, and the error structure and link function of the models were defined according to their distribution (e.g., Gaussian for risk-taking after log_10_-transformation and novelty avoidance; Gamma for aggression as it showed a strongly skewed distribution due to many individuals showing immediate attacks). To circumvent the problem posed by potential deviations from the chosen distributions and the effect of censored variables (e.g., five individuals that did not attack within 5 min were assigned a score of 301 for aggression), we also performed alternative analyses based on the rank-transformed values of the behavioral variables. To deal with the effect of the arbitrary ordering of males with the same aggression score, we repeated the ranking procedure multiple times (1000) and run the same models. Moreover, we also created bivariate categories based on the distribution of the variables (e.g., males with <5 sec latency scores were considered as immediate attackers, while males with >5 sec latency scores were hesitant attackers). These exercises targeting the distribution of the response variables gave qualitatively identical results to those of the starting models; thus, subsequently we focus on these.

The focal predictors in our models were the categorical variables containing the genotypes for SNP554 and SNP764. The model also included the standardized date of the behavioral observation to control for the potentially confounding effect of territory quality. In this approach, we assumed that arrival date (i.e., when the male was first seen on the field site) reflects territory quality, as males likely occupy territories in order of quality along their arrival to the breeding grounds (Kokko 1999). We also considered time of the day as a covariate to control for any within-day variation in behavior. Furthermore, age was entered as a categorical predictor as it can affect behavior (Garamszegi et al. 2006) and can reflect survival abilities that might potentially vary among *DRD4* genotypes. Year effects were handled by entering this variable as a random factor in the model, while we also used the identity of the decoy used in the behavioral test to control for the potential effect of similar response elicited by the same stimulus (we used 4–6 different stimulus birds in different years in a way that resulted in a balanced repetition between them). Initially, we considered both random intercepts and slopes to capture potential between-year differences in the focal association, but our model comparison exercises (see strategies below) revealed that the inclusion of random slopes into the model did not generally offer higher fit to the data (all *P*s > 0.7). Hence, we proceeded with the simpler models without random slope structures. We did not include additional variables in the model in order to avoid too many predictors relative to sample size. However, using an extended dataset (including behavioral measurements obtained from 2007–2013), we tested whether the response variables and the *DRD4* genotypes were related to male size, male condition, and the size of the two sexually selected plumage traits (wing patch size and forehead patch size). These analyses showed that none of these variables describing male quality affected strongly our focal variables (Table 1); thus, we can safely disregard them.

**Table 1:** The relationships between indices of male quality (tarsus length reflecting body size, size-corrected body mass reflecting body condition, and the size of the white forehead patch and wing patch reflecting the elaboration of two sexually selected plumage traits) and behavioral phenotypes and *DRD4* genotypes in the collared flycatcher

	Tarsus	Condition^1^	FPS^2^	WPS^3^
Novelty avoidance	*r* = −0.066, *P* = 0.422, *N* = 151	*r* = −0.134, *P* = 0.106, *N* = 146	*r* = 0.038, *P* = 0.642, *N* = 152	*r* = −0.059, *P* = 0.472, *N* = 151
Aggression	*r* = 0.009, *P* = 0.911, *N* = 167	*r* = −0.025, *P* = 0.747, *N* = 164	*r* = 0.023, *P* = 0.759, *N* = 170	*r* = 0.084, *P* = 0.279, *N* = 168
Risk-taking	*r* = −0.049, *P* = 0.530, *N* = 166	*r* = −0.018, *P* = 0.812, *N* = 162	*r* = 0.118, *P* = 0.127, *N* = 168	*r* = 0.088, *P* = 0.260, *N* = 166
SNP554^4^,^5^	*t* = −0.269, *P* = 0.789, df = 96.2	*t* = 0.628, *P* = 0.533, df = 53.8	*t* = 0.175, *P* = 0.861, df = 73.5	*t* = 0.315, *P* = 0.754, df = 77.6
SNP764^4^,^5^	*t* = −0.399, *P* = 0.692, df = 47.4	*t* = 0.321, *P* = 0.750, df = 29.6	*t* = −0.677, *P* = 0.503, df = 38.3	*t* = 0.851, *P* = 0.400, df = 38.4

Based on methods described in Peig and Green (2009, 2010).

Forehead patch size (height × width).

Age-corrected wing patch size, as described in Török et al. (2003).

Rare genotypes excluded.

Welch approximation to the degrees of freedom (assuming unequal variances).

Before interpreting the model outcomes, we performed numerous model diagnostics statistics to avoid misleading results based on statistical artifacts. We first checked assumptions about the distribution of residuals, that is, whether they were normally and homogeneously distributed. The visual inspection of the corresponding diagnostics plots (e.g., Q–Q plot and residuals plotted against fitted values) indicated no obvious deviations from these assumptions. Second, we examined issues about multicollinearity that might potentially lead to instable results and unreliable parameter estimates (Freckleton 2011). For this purpose, we calculated variance inflation factors (VIF, O'Brien 2007) to the standard linear model analog of each mixed model that was obtained after excluding the random effect (as this method is not available for mixed models). These analyses showed that collinearity among predictors is not a serious issue to consider further (VIFs < 2). Finally, we verified the absence of influential data points by excluding each of them one by one from the data and then contrasting the derived parameter estimates and fitted values against those that correspond to the model based on the full data. This jackknife procedure revealed no evidence for influential cases strongly affecting the interpretations of the model outcomes for novelty avoidance and risk-taking. For models on aggression, we identified two influential cases, but the exclusion of these data points did not affect the main conclusions of our study.

Parameter estimates were obtained by fitting models using maximum likelihood rather than restricted maximum likelihood (Bolker et al. 2009). To determine the strength of the focal relationship between behavioral phenotypes and *DRD4* genotypes, we performed likelihood ratio tests, in which we compared full models that included SNP554 or SNP764 as predictors with their restricted counterparts without the main predictor. The statistical significance of the focal predictor is then described by the probability function of the chi-square distribution at the degrees of freedom reflecting the difference between models in the number of parameters estimated (df = 1). Merely for illustration purposes, we provide such an estimation of statistical significance in the form of *P* value, but for biological interpretations, we focus on effect sizes (sensu Nakagawa and Cuthill 2007; Garamszegi et al. 2009a). Such interpretations make comparisons across traits and studies and also with repeatabilities more intuitive, and also eliminate the problems caused by multiple testing in a null hypothesis testing framework. To calculate effect sizes for each focal relationship, we computed Cramer's V, as V = square root (*χ*^2^/N), where *χ*^2^ is from the above likelihood ratio test and N is the total sample size (number of individuals). This index (but only at df = 1) is equivalent to the correlation coefficient *r*, thus the same guidelines can be applied for making inferences about the magnitude of the effect (Cohen 1988). Accordingly, we used the following benchmark from evolutionary ecology: *r *≈* *0.1 is a small effect, *r *≈* *0.3 is a medium effect, and *r *≈* *0.5 is a strong effect (Møller and Jennions 2002). If we used alternative approaches to calculate effect sizes, such as from estimated *t* values (Nakagawa and Cuthill 2007) as derived from the output of the full model, the results were highly similar to those we report below. Confidence intervals (95% CI) around effect sizes were obtained by parametric bootstrapping. For this, we simulated values for the response variable based on the estimated model parameters and then fitted the same model again to determine the effect of the *DRD4* trait on the simulated values of the behavioral trait. The effect size based on this bootstrap sample was determined as above. The whole procedure was repeated 1000 times to calculate the 5th and 95th quantiles as the confidence boundaries around effect sizes.

The statistical analyses were carried out in the environment of R (R Development Core Team 2007). For the mixed modeling, we used mainly the package lme4 (Bates et al. 2011), but for some types of distribution, we relied on MCMCglmm (Hadfield 2010). For a part of the model diagnostics, we relied on the VIF function available in package car (Fox and Weisberg 2011). For the parametric bootstrap and for the investigation of influential data points, we used our own scripts (developed by LZG, available upon request). For some verification, we also exploited some functions from packages languageR (Baayen 2007), pbkrtest (Halekoh and Højsgaard 2012), and influence.ME (Nieuwenhuis et al. 2012).

## Results

### Frequency distribution of *DRD4* genotypes within the population

For SNP554, “TT” homozygotes were extremely rare (CC: 134 individuals, CT: 62 individuals, TT: 4 individuals, Fig. 1), which corresponds to an allele frequency of *f*(T) = 0.14 and 1−*f*(T) = *f*(C) = 0.86. The population did not significantly deviate from Hardy–Weinberg equilibrium that can be predicted from these allele frequencies (*χ*^2^ = 4.938, *P*_simulated_ = 0.083). Similar calculations for SNP764 revealed allele frequencies of *f*(C) = 0.12 and *f*(A) = 0.88 (AA: 153 individuals, AC: 46 individuals, CC: 3 individuals, Fig. 1), and the sample showed genotype frequencies that did not violate expectations form the Hardy–Weinberg equilibrium (*χ*^2^ = 0.226, *P*_simulated_ = 0.933).

Sexes and age categories showed no significant differences in their genotypes (sex, SNP554: *χ*^2^ = 1.765, *P*_simulated_ = 0.469; SNP764: *χ*^2^ = 0.743, *P*_simulated_ = 0.794; age, SNP554: *χ*^2^ = 4.632, *P*_simulated_ = 0.082; SNP764: *χ*^2^ = 0.021, *P*_simulated_ = 0.999). Tests for independence of the genetic variation at the two SNPs revealed that they are correlating (including all genotypes: *χ*^2^ = 111.1, df = 4, *P*_simulated_ < 0.001, excluding rare genotypes, that is, “TT” for SNP554 and “CC” for SNP764: *χ*^2^ = 40.34, df = 1, *P*_simulated_ < 0.001). This pattern showed that the occurrence of the genotype combinations of SNP554CC × SNP764AA and SNP554CT × SNP764CA was more common than would be expected by chance, indicating that there is some linkage disequilibrium between the C allele of SNP554 and the A allele of SNP764 (a correlation between genotypes is equivalent with an effect size of Cramer's V = 0.454).

### The relationship between behavioral traits and between behavior and *DRD4* polymorphism

In the whole sample spanning over 7 years, there was an evidence for a weak to medium, and consistently positive correlation between behavioral traits (novelty avoidance–aggression: *r *=* *0.170, *N* = 188, 95% CI = 0.028/0.306, *P *=* *0.019; novelty avoidance–flight initiation distance: *r *=* *0.126, *N* = 182, CI = −0.020/0.267, *P *=* *0.091; aggression–flight initiation distance: *r *=* *0.310, *N* = 221, CI = 0.186/0.425, *P *<* *0.001; see also Garamszegi et al. 2009b, 2012b). This pattern was also prevalent in the subsample of males, for which we had genetic information (novelty avoidance–aggression: *r *=* *0.202, *N* = 51, 95% CI = −0.078/0.452, *P *=* *0.152; novelty avoidance–flight initiation distance: *r *=* *0.048, *N* = 53, CI = −0.225/0.314, *P *=* *0.730; aggression–flight initiation distance: *r *=* *0.387, *N* = 52, CI = 0.128/0.597, *P *=* *0.005).

**Figure 3:**
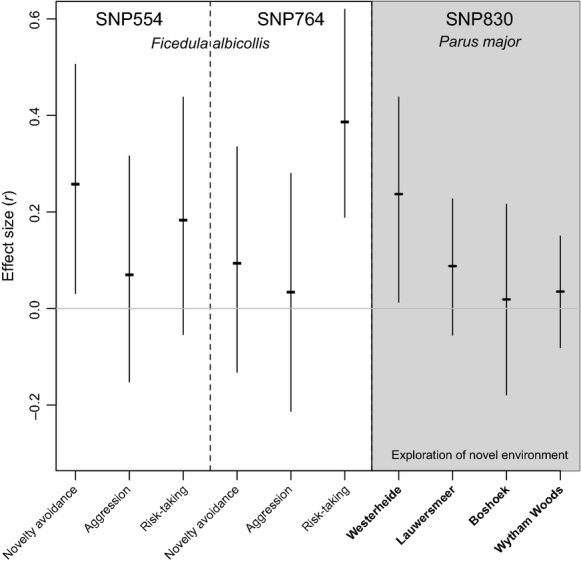
The comparison of effect sizes (Cramer's V representing *r* effect size calculated from the *χ*^2^ statistics of the appropriate likelihood ratio test) found in the present study in a Hungarian collared flycatcher population with those that were detected in another study testing similar predictions in four wild populations of the great tit, *Parus major* (Korsten et al. 2010). Disregarding the direction of the effect, unsigned effect sizes are shown (horizontal lines). The 95% CIs (vertical lines) for the collared flycatcher are based on bootstrapping (see Methods and Table 2), while for the great tit are based on the approximation method through standard errors (see Nakagawa and Cuthill 2007).

We compared behavioral scores of males monitored during the courtship period across *DRD4* genotypes. In this subsample, the rare “TT” homozygote for SNP554 and the “CC” homozygote for SNP764 were not represented; thus, the comparisons are formally based on two genotype groups per SNP. We controlled for the effect of potentially confounding factors in a mixed model design. The results as revealed by this approach are given for each of the two tested genetic markers and for each behavioral trait in Table 2. The strongest and most obvious pattern was the relationship between SNP764 genotype and risk-taking behavior, which showed that heterozygote individuals take lower risk when a potential predator is approaching (i.e., flee at higher distance) than individuals having the common allele “A” in homozygote combination (Fig. 2B). The corresponding effect size was almost *r *=* *0.4, and the associated confidence intervals indicated effect sizes representing medium to strong effects (Fig. 3). A considerable effect size (i.e., with a bootstrap confidence interval falling into the positive direction) was also detected for the relationship between SNP554 polymorphism and novelty avoidance indicating a tendency for heterozygote individuals being more cautious in the presence of novelty than “CC” homozygotes (Figs. 2A and 3). Although, we adopt the effect size approach for the interpretation of the results, for readers preferring the null hypothesis testing framework, we report that only the former relationship exceeded the significance threshold after correcting for multiple testing by the use of Bonferroni adjustment on *P* values (*P*_critical_ = 0.0083).

**Table 2:** The relationships between behavioral traits (novelty avoidance, aggression, and risk-taking) and *DRD4* polymorphism as shown at two SNPs (SNP554 and SNP764) while controlling for the potentially confounding effects of the date of arrival (reflecting territory quality), the time of behavioral assay, age at sampling, and the hierarchical structure of data caused by repetitions within year and stimulus bird in a wild population of the collared flycatcher. Results from generalized linear mixed models, in which behavioral variables were response variables and genotypes were the main predictors. The confounding variables were entered as control variables (date, time, age) or random effects (year, decoy identity). Significance levels (*P*) and effect sizes (Cramer's V reflecting *r*) were only calculated for the variables (*DRD4* genotypes) that are of relevance for the objectives of the study. These originated from the corresponding likelihood ratio test that compared the model fit of the full model and the reduced model after excluding the focal variable. The 95% CIs around effect sizes originated from the parametric bootstrap performed on data simulated according to the model's predictions

Model	Estimate (SE)	*r* effect size (95% CI_lower_/CI_upper_)	*P*_likelihood ratio_
Novelty avoidance			
Intercept	602.84 (239.52)		
SNP554 (CT)	64.49 (33.42)	0.258 (0.031/0.506)	0.058
Date	77.52 (62.56)		
Time	−674.17 (238.10)		
Age (juv)	48.54 (38.14)		
Novelty avoidance			
Intercept	638.30 (245.96)		
SNP764 (CA)	25.58 (36.57)	0.094 (−0.132/0.335)	0.491
Date	78.10 (64.70)		
Time	−693.27 (244.82)		
Age (juv)	39.92 (38.95)		
Aggression			
Intercept	−119.05 (558.14)		
SNP554 (CT)	39.63 (76.82)	0.070 (−0.152/0.316)^1^	0.608^2^
Date	44.03 (151.55)		
Time	546.58 (545.76)		
Age (juv)	139.45 (95.66)		
Aggression			
Intercept	−79.98 (556.22)		
SNP764 (CA)	−20.21 (80.88)	−0.034 (−0.280/0.213)^2^	0.804^2^
Date	42.26 (152.01)		
Time	528.41 (545.51)		
Age (juv)	132.01 (95.21)		
Risk-taking			
Intercept	0.836 (0.522)		
SNP554 (CT)	0.096 (0.073)	0.183 (−0.054/0.438)	0.191
Date	0.053 (0.127)		
Time	0.139 (0.520)		
Age (juv)	0.032 (0.091)		
Risk-taking			
Intercept	0.849 (0.491)		
SNP764 (CA)	0.214 (0.075)	0.387 (0.189/0.620)	0.006
Date	0.053 (0.120)		
Time	0.104 (0.490)		
Age (juv)	0.025 (0.085)		

When entering aggression as a bivariate variable (“immediate” or “hesitant” attacker) and using binomial error distribution: *r *=* *0.142 (−0.133/0.397), *P *=* *0.303.

When entering aggression as a bivariate variable (“immediate” or “hesitant” attacker) and using binomial error distribution: *r *=* *0.166 (−0.109/0.418), *P *=* *0.228.

## Discussion

We identified two SNPs of the *DRD4* orthologue in a wild collared flycatcher population. The genotype frequencies were comparable with the unequal proportion of genotypes detected in other bird species for other *DRD4* orthologues (Fidler et al. 2007; Korsten et al. 2010; Gillingham et al. 2012; Carvalho et al. 2013; but see Kluen et al. 2012 for more or less equal allele frequencies). We related this genetic variation to phenotypic variations in behavior. Compared to previous studies on similar topic, our results are novel in regard that (1) we focused on behaviors other than novel environment exploration; and (2) we screened more than one behavior that positively correlated with each other potentially reflecting behavioral consistency at the between-trait level, that is, a behavioral syndrome. Furthermore, an important aspect of our study is that, in contrast to the laboratory or captivity tests performed in previous studies, our behavioral tests are genuine field tests offering stronger implications for the genetic basis of behavior under natural conditions. We note that similarly to other studies on related topic, our results are merely correlative and cannot be used to make strong inferences about causal relationships. Below, we provide some possible explanations for the detected patterns.

The most striking result of our study was that genetic polymorphism at the SNP764 was related to the phenotypic variation in risk-taking behavior, as estimated by flight initiation distance. In our statistical framework, such a relationship could be best described as an effect with medium size. However, given the limitations of our data, which define a given confidence around this approximation, we remain uncertain about the exact whereabouts of the true effect along the continuum from small to strong effects. In any case, such an effect has a biological importance for at least two reasons. First, behaviors are known to be under the control of multiple genes leaving very small effects for each particular gene (Tschirren and Bensch 2010). Second, behaviors can be described by modest repeatability (Bell et al. 2009; Garamszegi et al. 2012a), and heritability (Drent et al. 2003; Quinn et al. 2009), which also sets an upper limit for the strength of the detected correlation (Spearman 1904; Falconer and Mackay 1996). In light of this premise, in an earlier study of the studied population, in which we had multiple data on the same behaviors from the same season, we found that flight initiation distance has a repeatability around 0.6 that is higher than that of the other behaviors (Garamszegi et al. 2012b). This higher within-individual consistency may explain why we detected the strongest relationship for this trait, and not for the others with lower repeatability (e.g., *r *=* *0.31 for novelty avoidance and *r *=* *0.38 for aggression).

It is informative to compare the effect sizes we identified here with those that were discovered in other studies under a similar hypothetical framework (Fig. 3). Korsten et al. (2010) estimated the strength of the relationship between *DRD4* polymorphism and exploration behavior in four European population of the great tit, and found that the strength of relationship between these traits can vary on a spatial scale. Exploration reflects the activity of movements in a novel environment that could be considered as an inverse estimate of novelty avoidance (Réale et al. 2007). Furthermore, the exploration of novel environment is repeatable over time (Quinn et al. 2009; Dingemanse et al. 2012) and also correlates with aggression and risk-taking in great tits (van Oers et al. 2004; Groothuis and Carere 2005; Hollander et al. 2008). Such exploration scores at the phenotypic level varied systematically with *DRD4* polymorphism at the genetic level estimated at SNP830 in a Dutch great tit population, but not in three other locations (Fidler et al. 2007; Korsten et al. 2010). The statistical evidence about the existence of the link between *DRD4* genotype and behavior in one population embodies an effect size of an immediate magnitude (*r* ∼ 0.3) with a confidence interval that highly overlaps with some of the effect sizes we detected in this study (Fig. 3). Although our sample size was smaller causing an increase in uncertainty, it seems likely that the strength of the relationship between SNP554 and novelty avoidance, between SNP764 and risk-taking, and even between SNP554 and risk-taking in the collared flycatcher is comparable with that of the relationship between SNP830 and exploration score in the great tit. Hence, considering the error rates that are defined by within-individual variation in behavior and also the effect of other genes, we suggest that the relationships we detected between *DRD4* and behavior have biological significance. Altogether, it seems that behaviors that involve “fear” components (i.e., response to novel object, novel environment, or potential predator that can put individuals in a life-threatening situation) have a higher potential to be associated with *DRD4*. This contextual overlap is also in line with the fact that novelty seeking is the most important behavioral correlate of the genetic region (Kluger et al. 2002; Schinka et al. 2002; Reif and Lesch 2003; Ebstein 2006).

However, the main difference between these two sets of results was that in the collared flycatcher, the heterozygote genotypes show generally more risk aversion (i.e., longer latencies to resume courtship activity and larger flight initiation distances) than the homozygotes for the common alleles (“CC” for SNP554 and “AA” for SNP764, Fig. 2). On the other hand, in the great tit, the heterozygotes can be characterized by their higher exploration scores signifying higher risk-taking than the more frequent homozygote genotype at SNP830 (Fidler et al. 2007; Korsten et al. 2010). Whether there is a cause-and-effect connection between the detected allele frequencies and the fitness consequences of risk-taking behavior remains an open question. In a previous 1-year study, we were able to demonstrate that singing male flycatchers reveal their risk-taking during their singing behavior and such behavior can influence the outcome of mating success (Garamszegi et al. 2008). Therefore, if such female preference for risk-taking males was generally acting in the population, this would also affect the frequencies of the associated genes leading to the preponderance of the genotypes that are linked with higher risk-taking and the dilution of genotypes that accompany less risk-taker behavior. The detected proportions of genotypes fulfill this scenario. However, we could not obtain statistical evidence for the deviations from the Hardy–Weinberg equilibrium that would prove the existence of such evolutionary influences through nonrandom mating or selection. Perhaps, fluctuating and unpredictable environmental conditions (e.g., change in food supply, predation pressure, or competition level for breeding opportunities) may make the fitness benefits of risk-taking to vary across years (Dingemanse et al. 2004). As such, if higher risk-taking involves mating benefits in 1 year but it is costly in other years, it could render allele and genotype frequencies to remain constant in the long term within the population (spatio-temporal variation in selection). We note that we could not detect year-specific effects by investigating random slope regressions over the 3-year study period. It is also plausible that the investigated behaviors are linked to other genes and the investigated SNPs are linked to other behaviors, thus a complex trade-off mechanism regulating the entire multigene/multibehavior system. Accordingly, we found that variations at the two *DRD4* SNPs are not independent of each other.

In any case, inferences about the underlying genetic model for the impact of *DRD4* polymorphism on consistent behavior remain merely speculative. It is currently unknown how genetic variation at the investigated SNPs is translated into variation at the phenotypic level, because the nucleotide substitutions involved are synonymous that do not alter amino acid sequence of the decoded protein (Fidler et al. 2007). However, this phenomenon does not necessarily mean that the SNPs are nonfunctional *per se*. The effects of such synonymous mutations on transcription, splicing, and mRNA stability as well as linkage disequilibrium (a link with other nonsynonymous polymorphism) have been suggested as potential mechanisms that may mediate a functional relationship between the *DRD4* SNPs and behavior (Duan et al. 2003; Chamary et al. 2006; Flisikowski et al. 2009). We note that a recent study shows that linkage disequilibrium may only play a minor role, and population-specific adaptive histories may mediate the locally detected associations between genetic polymorphism and exploration (Mueller et al. 2013). However, the association found between SNP554 and SNP764 in our species indicates linkage disequilibrium or other functional links operating between the two genetic regions.

If the detected associations truly reflect the importance of the genetic control of behaviors, they could have important implications for the evolution of behavioral syndromes. The constraint hypothesis posits that the common physiological background can cause the governed behavioral traits to covary (Bell 2005). For example, the pleiotropic effects of regulatory hormones or genes can trigger an entire suite of behaviors, and as a result, functionally independent behaviors become evolutionary nonindependent due to the shared control machinery. Such strong genetic associations can set up important constraints for the evolvability of traits by preventing certain trajectories of evolutionary responses available to them (Dochtermann and Dingemanse 2013). On the other hand, looser mechanistic links between behaviors (e.g., due to environmental constraints) permit easier decoupling that further opens a horizon for more flexible evolutionary responses (Sih et al. 2004). The associations between the two SNPs and between behaviors, and the fact that those traits that have “fear” components tend to show stronger relationships with *DRD4* polymorphism (Table 2 and Fig. 3), may imply that, at least, partially similar or overlapping control mechanisms are in effect.
